# Intravascular Shockwave Lithotripsy for the Treatment of Severe Peripheral Arterial Disease: A Single-Centre Experience

**DOI:** 10.7759/cureus.78149

**Published:** 2025-01-28

**Authors:** Shanga Salihi, Omar Suleiman, Bilal Afzal Tarar, Fitoon Yaldo, Mojahid Najem

**Affiliations:** 1 Vascular Surgery, London Northwest University Healthcare NHS Trust, London, GBR; 2 General Surgery, Royal Devon Healthcare NHS Foundation Trust, Barnstaple, GBR; 3 Cardiothoracic Surgery, University Hospitals Bristol and Weston NHS Foundation Trust, Bristol, GBR

**Keywords:** chronic limb threatening ischemia, critical limb ischemia, intravascular shockwave lithotripsy, peripheral arterial disease, shockwave angioplasty

## Abstract

Background

Chronic limb-threatening ischemia (CLTI) is the most severe manifestation of peripheral arterial disease. Various revascularization techniques are employed to treat peripheral arterial disease. Intravascular shockwave lithotripsy (IVL) is a relatively new procedure for the treatment of calcific lower limb peripheral arterial diseases (PAD).

Objectives

To assess the effectiveness and safety of shockwave lithotripsy in patients with severe PAD through an evaluation of limb salvage rate and patient survival.

Methods

A retrospective study of all patients treated with shockwave lithotripsy between November 2019 and June 2024 was performed. The primary outcome was amputation-free survival and secondary outcomes were potential complications of IVL (thrombo-embolization, perforation, and restenosis). Patients were followed up in the clinic and assessed both clinically and with a duplex scan at three months.

Results

A total of 38 patients were included in the study. The median age was 71 years; 28 were males. Among the patients, 28 (73.68%) were diabetic, 4 patients (10.53%) were current smokers, 10 patients (26.32%) were ex-smokers, and 24 patients (63.16%) were non-smokers. According to the Rutherford classification of PAD, 33 of the 38 patients were in stages 4-6. Only five patients were stage 3. Total number of treated arteries was 47. Treated lesions were 49% in the superficial femoral artery (SFA), 36% in the popliteal artery, 8% in the common femoral artery (CFA), 4% received treatment of the iliac arteries, and 2% of the posterior tibial artery. All treated arteries showed improved angiographic results. Four patients (10.53%) developed distal embolization. No perforation was recorded, and no significant flow-limiting dissection was recorded to require treatment. At the three-month follow-up, imaging revealed improvement in 58% of patients while 5% showed no improvement. Notably, follow-up imaging was not conducted in 37% of patients due to evident clinical improvement such as ulcer healing, palpable pulses, and the presence of Doppler signals. Seven patients required reintervention within three months after the initial IVL operation and 4 patients got revascularized after this period resulting in 11 patients requiring revascularisation after the initial operation. Amputation-free survival was 79% (30 patients).

Conclusion

Shockwave lithotripsy is associated with a high limb salvage rate and low complication rate. Further research is needed into long-term effectiveness and the role of shockwave treatment as an adjuvant to traditional revascularization techniques of patients with CLTI and short-distance claudicants.

## Introduction

Intravascular shockwave lithotripsy (IVL) is an innovative therapeutic approach for treating lower limb peripheral arterial disease (PAD), particularly in cases involving calcified arterial lesions. The development of this technology is rooted in the application of shockwave therapy, which has been successfully used in urology for years, specifically for breaking down kidney stones. IVL harnesses the same principles of focused shockwave generation to fragment calcified plaque in peripheral arteries, thereby facilitating improved blood flow and reducing symptoms associated with PAD [1].

The application of IVL in vascular treatment emerged in the early twenty-first century, with initial studies demonstrating its efficacy in disrupting calcium deposits that often limit the effectiveness of traditional endovascular interventions such as balloon angioplasty and stenting [2]. The unique mechanism of action allows the shockwaves to penetrate deep into the target tissues while minimizing damage to the surrounding vascular structures. Clinical studies have indicated that IVL can enhance procedural success rates, prolong patency, and improve overall limb outcomes in patients suffering from critical limb ischemia and intermittent claudication [3].

Potential benefits of IVL include its minimally invasive nature, reduced risk of downstream embolization, and the ability to treat complex calcified lesions that are challenging to manage with conventional techniques. Furthermore, patients may experience quicker recovery times and less procedural discomfort as compared to more invasive surgeries [4]. Despite its advantages, IVL is not without complications. The risks may include transient pain, vascular injury, and, in rare cases, thromboembolic events, highlighting the need for careful patient selection and procedural expertise [5].

Intravascular shockwave lithotripsy represents a promising advancement for managing lower limb peripheral vascular disease. With its ability to safely and effectively address calcified lesions, IVL is poised to significantly impact the landscape of vascular interventions, aligning with the ongoing pursuit of improved patient outcomes in PAD management [6].

This study was carried out to assess the efficacy and safety of shock wave lithotripsy in the management of severe PAD in a single centre within a year of the procedure. An assessment was made of the limb salvage rate, overall survival, and rates of major and minor amputation following lithotripsy. The study seeks to determine if lithotripsy can at least be an option or complementary intervention for the patients with PAD of Rutherford classes 3, 4, 5, and 6.

## Materials and methods

Study design

This study focuses on the utilization of shockwave lithotripsy in the treatment of severe PAD in patients diagnosed with chronic limb-threatening ischemia (CLTI) or short-distance claudication. A retrospective approach was adopted, retrieving patient data including re-intervention and amputations from the electronic medical records of 38 consecutive patients treated between November 2019 and June 2024. Every patient who received shockwave as part of PAD treatment was included. A detailed inline review and analysis of angiographic images were performed using the institution's digital imaging viewer system. The Peripheral Arterial Calcium Scoring System (PACSS) score was calculated prior to the intervention and was independently reviewed by two consultant vascular surgeons, who demonstrated complete agreement with no discrepancies observed. In addition to this, the Global Anatomic Staging System (GLASS) Classification as well as the Trans-Atlantic Inter-Society Consensus (TASC) classification was recorded for each patient. Data were collected using an Excel sheet (Microsoft Corporation, Redmond, WA, US). Statistical analysis was done using the analysis module of Microsoft Excel software, and amputation-free survival analysis was performed using SPSS v29 (IBM Corp., Armonk, NY, US). The study was subjected to review and approval by the Institutional Review Board to ensure adherence to ethical and regulatory standards.

Patients

The study included all adult patients diagnosed with CLTI or short-distance claudication, who underwent shockwave lithotripsy for calcified peripheral arterial lesions. Patients meeting these eligibility criteria were identified retrospectively, and their medical records were reviewed for inclusion in the analysis.

Intervention

Shockwave lithotripsy, or IVL, was employed in this patient to treat calcified plaques within peripheral arteries. The purpose of the procedure was to disrupt calcified deposits in the arterial walls using high-intensity sound waves. This disruption facilitates the dilatation of the vessel lumen during lithotripsy angioplasty, thereby enhancing the delivery of stents or angioplasty balloons. The ultimate aim is to restore blood flow to the affected limb and improve tissue perfusion.

**Figure 1:**
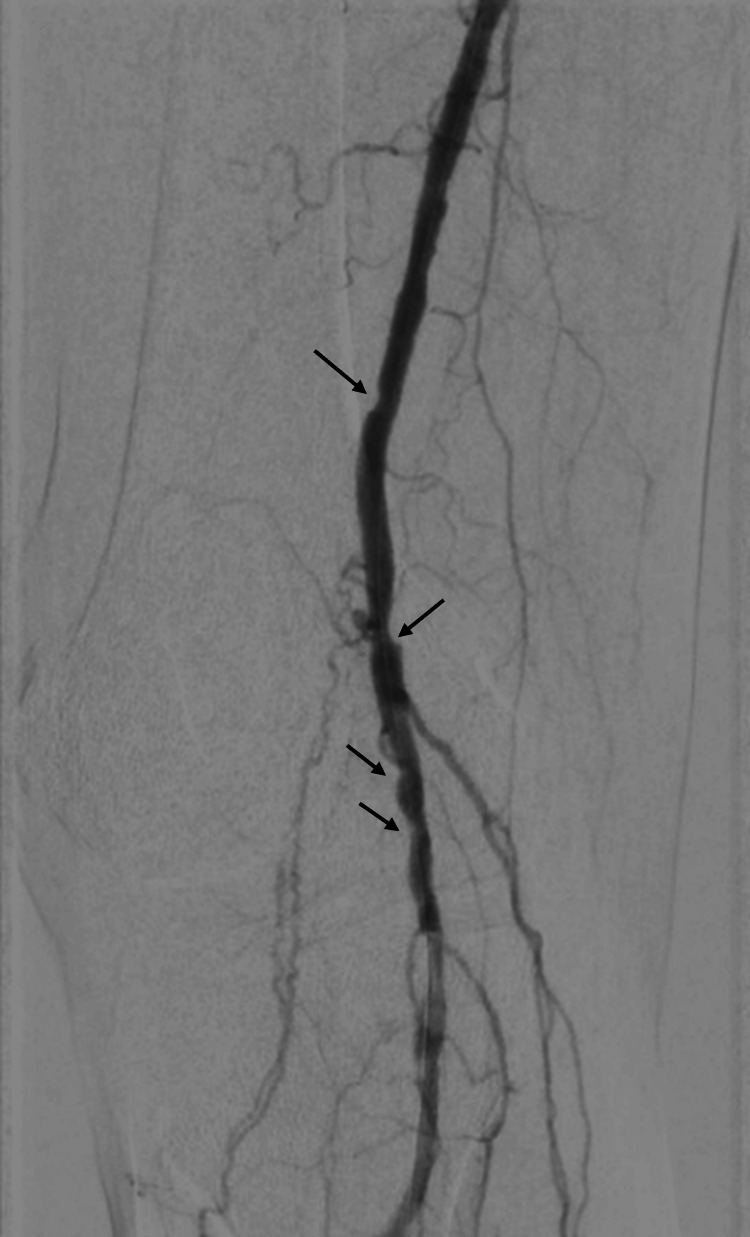
Distal SFA and popliteal artery showing calcified lesions prior to the use of shockwave lithotripsy SFA: superficial femoral artery

The lithotripsy system used in this study was the shockwave lithotripsy device from Shockwave Medical, Inc. (Santa Clara, CA, US). This system delivers acoustic pulses directly to the calcified lesions through a catheter-based device. A sample pre-intervention image is depicted in Figure 1.

During the procedure, patients were monitored for immediate complications, including vessel dissection, perforation, restenosis, and embolic events such as trash foot.

**Figure 2:**
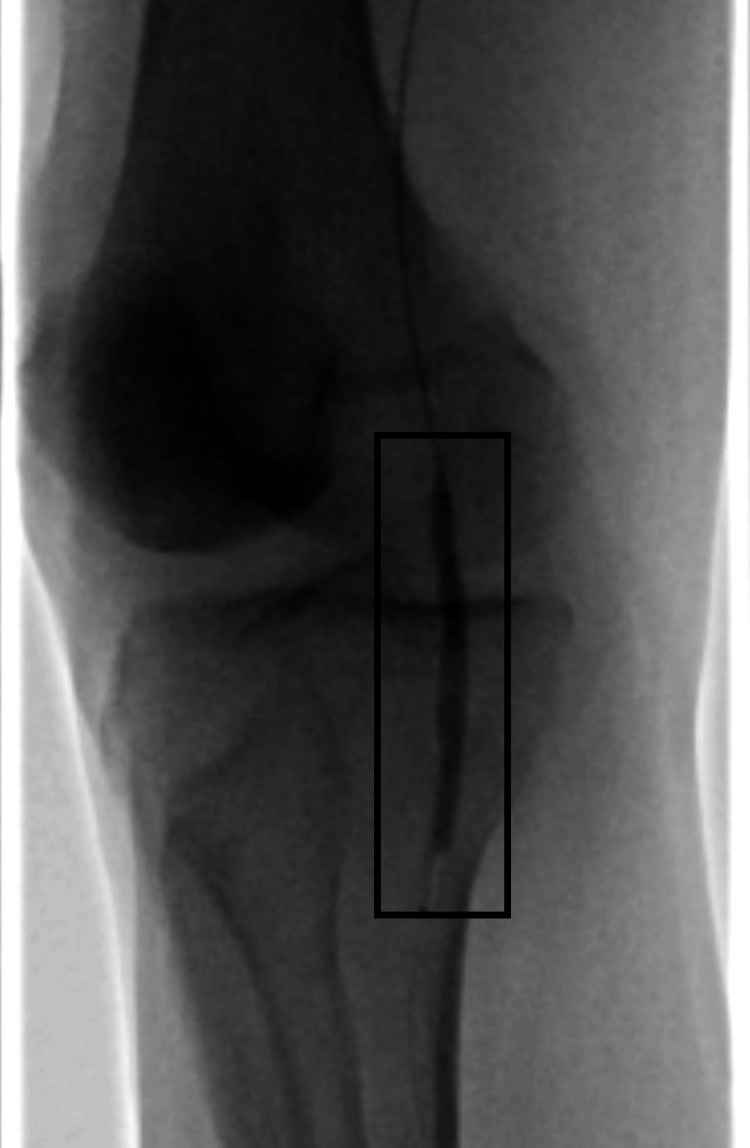
Inflated balloon shockwave in the popliteal artery segment The rectangle highlights the shockwave balloon being inflated in a segment of the popliteal artery.

Figure 2 shows the popliteal segment while the shockwave balloon is inflated.

Post-operative assessments were carried out to document and address any procedural complications that arose both immediately and after one year.

**Figure 3:**
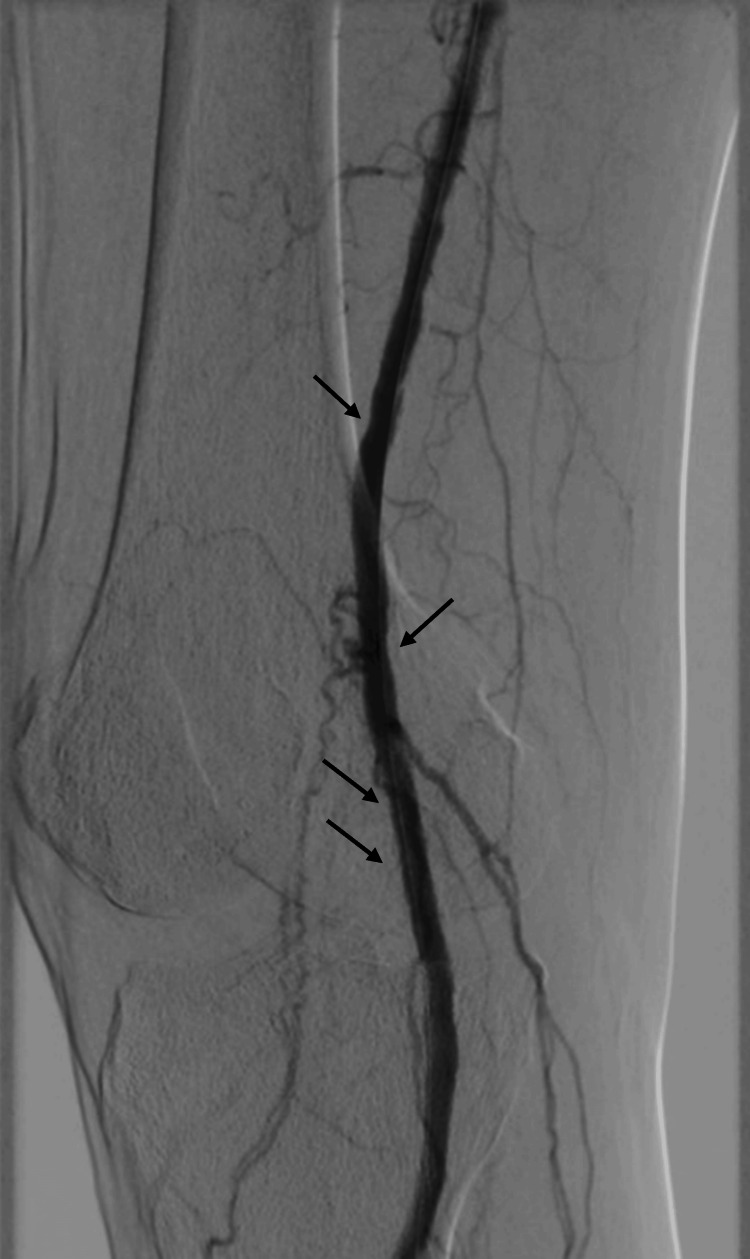
Popliteal and SFA segments after the treatment SFA: superficial femoral artery

Figure 3 shows the superficial femoral artery (SFA) and popliteal artery after the use of shockwave.

## Results

Demographics

A total of 38 patients were included in the study of which 33 were CLTI and 5 were short-distance claudicant. Baseline clinical characteristics are shown in Table 1.

**Table 1:** Baseline characteristics of patients

Age Range (years)	46 – 86 y (mean age 71y)
Gender Distribution (M : F)	28 : 10 (males: 74%)
Diabetes History	73.6%
Smoking History	42%

In total 47 vessels were treated, the superficial femoral artery was the most treated artery followed by the popliteal artery. Some of the patients had more than one arterial segment treated with IVL. Table 2 shows the distribution of the treated arteries.

**Table 2:** Target vessel distribution

Vessel	Target vessels treated
Iliac Artery	2
Common Femoral Artery	4
Superficial Femoral Artery (SFA)	23
Popliteal Artery	17
Posterior Tibial Artery	1

Patient images were classified according to the peripheral artery calcification scoring system (PACSS) [7]. The majority of treated vessels were grade four on the PACSS score as shown in Table 3. When multiple target vessels are treated, the worst grade is recorded. 

**Table 3:** PACSS classification PACSS: peripheral artery calcification scoring system

Grade	Score
Grade 0	0
Grade 1	3
Grade 2	4
Grade 3	6
Grade 4	25

Primary outcome

**Figure 4:**
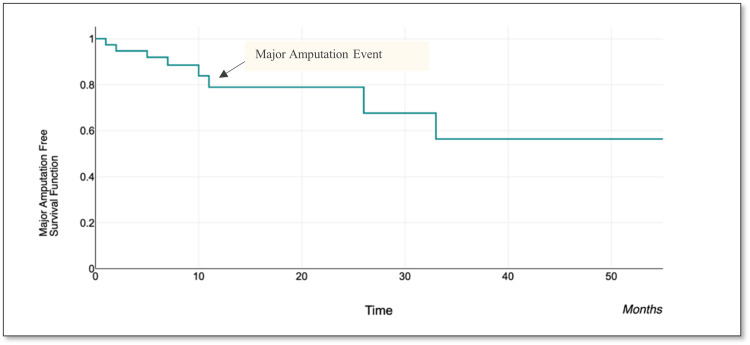
Amputation-free survival

Overall mortality during the study follow-up period was 18.4% (seven patients). There was one case of 30-day mortality with the others occurring at later periods and unrelated to the procedure. In this cohort, only 1 patient (2.6%) required major amputation 11 months after undergoing treatment. This confirms the significant ability to preserve limbs using shockwave lithotripsy. Planned minor amputations below the level of the ankles were required for seven patients (18%). Figure 4 is the Kaplan-Meier curve illustrating the amputation-free survival in this study.

Secondary outcomes

Out of 38 patients, 4 patients developed distal embolization. No perforation was recorded and no significant dissection was recorded.

After the initial operation, 11 patients required revascularization over the 10 months following the procedure, of which 7 patients required revascularization in the same limb within 3 months from the initial operation.

## Discussion

CLTI is a complex entity in vascular surgery and when left untreated, it can jeopardize the life of the patient through amputation or death. Intravascular shock wave lithotripsy or IVL is an advanced approach to management. This technology is ideal for patients who are contraindicated for open interventions or cannot be treated with conventional endovascular interventions. IVL functions by applying sound energy pressure waves through the vessel walls and is directed to mechanically break the calcium deposits in the arteries to allow vessel dilation during balloon angioplasty for revascularization. Many studies have established the effectiveness of this modality for PAD and CLTI patients particularly [8].

The advancements in treatment options, when applied collaboratively and tailored to individual patient characteristics, may contribute to reducing the rate of major amputations [9]. In the present study, with a median follow-up of 11 months (range 4 - 58 months), the major amputation rate of 2.6% is comparable to other studies [10].

Similarly, the one-year survival rate of 78% achieved in the present study can be said to be in close agreement with other studies evaluating lithotripsy and other revascularization techniques [10]. This adds evidence to the increasing relevance of IVL in contemporary vascular interventional procedures. The inclusion of IVL in current standard treatment regimens is a major step forward in treating PAD and CLTI, thereby providing hope for improved patient prognosis and decreased mortality [9].

The study also examines embolization outcomes, emphasizing the tailored intraoperative management for each case. While embolic events were observed, the absence of clinical symptoms postoperatively is reassuring. It is, however, crucial to acknowledge that embolization cannot be solely attributed to IVL, given the concomitant use of plain old balloon angioplasty (POBA) and other endovascular techniques. The procedural complexity in cases with fresh or soft plaques reinforces the need for careful patient selection to mitigate embolic risks. These findings highlight the necessity of integrating IVL with other strategies to ensure optimal safety and efficacy in high-risk patients. Each patient was managed intraoperatively with tailored interventions. The first patient underwent multiple complex procedures, including distal puncture, to ultimately restore inflow. For the second patient, the shockwave device was used in the presence of a soft plaque, which was managed intraoperatively with aspiration and stenting. The third patient presented with trifurcation disease and SFA calcification, which was addressed through balloon angioplasty as part of the treatment strategy. The fourth patient developed microemboli that could not be retrieved.

Efficiency of lithotripsy

The results of the current study demonstrate that shockwave lithotripsy yields benefits over conventional limb salvage strategies for CLTI. First, the described technique is minimally invasive and can be performed under local anaesthesia, making it possible to avoid risks to patients who are at high risk for general anaesthesia or surgical treatment. Considering the capacity of lithotripsy to cleave calcified plaques, favourable vessel enlargement and stent deployment are facilitated. The finding of a high limb salvage rate also upholds our conclusion that lithotripsy results in improved blood circulation and reduced invasive surgical requirements. Follow-up scans at three months showed improvement in 58% of patients while 5% had no improvement and 37% were clinically improving with either wound healing, palpable distal pulses, or good Doppler signals and were not scanned, which is 95% of the patients collectively in comparison to 30 days follow-up result from the Disrupt PAD III trial, which shows 65.8% procedural success rate [12]. Furthermore, they showed less occurrence of restenosis in comparison to the use of a standalone percutaneous transluminal angioplasty [11-13].

Safety profile

The majority of patients tolerated the procedure without major complications such as vessel perforation or dissection. The distal embolization rate was 4 out of 38 patients (10.5%), which may not be solely attributed to the shockwave use, as there is concomitant usage of POBA that can be the cause of trash foot, which is consistent with findings from other studies on endovascular interventions for PAD [8]​.

Furthermore, the safety profile of lithotripsy is confirmed by the absence of vessel perforation and major bleeding that is characteristic of other related endovascular procedures such as atherectomy. The result of this study implies that lithotripsy is not only an efficient treatment approach but also safe for the treatment of heavily calcified arterial lesions in patients with CLTI [13]. IVL represents a promising and new method for addressing calcification in peripheral arteries. The current research offers evidence affirming the safety and efficacy of IVL. Numerous studies have demonstrated enhanced luminal gain, vascular diameter, and less mean residual stenosis, alongside improvements in clinical indices for patients whose vessels were prepared with intravascular lithotripsy [14,15].

Limitations

While the findings are promising, several limitations must be acknowledged. The small sample size (n = 38) restricts the generalizability of the findings, and the retrospective, single-center design introduces potential selection bias. The lack of a randomized control group limits direct comparisons with other revascularization techniques. Additionally, the median follow-up of 11 months does not provide long-term insights into restenosis or sustained limb salvage. The heterogeneity of treated lesions and the concomitant use of POBA complicates the isolation of IVL’s specific effects.

## Conclusions

Shockwave lithotripsy is recognized as a well-tolerated and effective treatment, providing a minimally invasive alternative to traditional endovascular techniques with promising outcomes. Our study highlights a high rate of amputation-free survival, further reinforcing its safety profile and making it an attractive option for managing patients with severely calcified arterial lesions.

Further research is essential to assess the long-term effectiveness of shockwave lithotripsy and to investigate its role as an adjunct to traditional revascularization techniques in patients with CLTI and short-distance claudication. While the initial results are encouraging, prospective multicenter studies are needed to evaluate the broader applicability and long-term benefits of this technique. Such studies will help clinicians refine its integration into existing treatment protocols.
